# Regulatory roles and mechanisms of alternative RNA splicing in adipogenesis and human metabolic health

**DOI:** 10.1186/s13578-021-00581-w

**Published:** 2021-04-01

**Authors:** Yunqi Chao, Yonghui Jiang, Mianling Zhong, Kaiyan Wei, Chenxi Hu, Yifang Qin, Yiming Zuo, Lili Yang, Zheng Shen, Chaochun Zou

**Affiliations:** 1 Department of Endocrinology, The Children’s Hospital, School of Medicine, Zhejiang University, Hangzhou, 310052 Zhejiang China; 2 Department of Genetics, Yale University School of Medicine, New Haven, CT 06520 USA

**Keywords:** Alternative splicing, Adipogenesis, Splicing factor, Obesity, Metabolic disorders

## Abstract

Alternative splicing (AS) regulates gene expression patterns at the post-transcriptional level and generates a striking expansion of coding capacities of genomes and cellular protein diversity. RNA splicing could undergo modulation and close interaction with genetic and epigenetic machinery. Notably, during the adipogenesis processes of white, brown and beige adipocytes, AS tightly interplays with the differentiation gene program networks. Here, we integrate the available findings on specific splicing events and distinct functions of different splicing regulators as examples to highlight the directive biological contribution of AS mechanism in adipogenesis and adipocyte biology. Furthermore, accumulating evidence has suggested that mutations and/or altered expression in splicing regulators and aberrant splicing alterations in the obesity-associated genes are often linked to humans’ diet-induced obesity and metabolic dysregulation phenotypes. Therefore, significant attempts have been finally made to overview novel detailed discussion on the prospects of splicing machinery with obesity and metabolic disorders to supply featured potential management mechanisms in clinical applicability for obesity treatment strategies.

## Introduction

Alternative splicing (AS) is a crucial post-transcriptional mechanism to reprogram gene expression profiles and expand transcriptomic and proteomic diversity in eukaryotic organisms [1, 2]. This highly dynamic process alternatively removes introns from a transcribed precursor messenger RNA (pre-mRNA) and combines various exons to facilitate one single protein-coding gene to finally form variable forms of mature mRNA species [2]. About 95% of human multi-exon genes can undergo splicing process [3]; nevertheless, a large majority of alternatively spliced variants may not be translated into proteins, so very few annotated alternative isoforms can be detected in large-scale proteomics studies [4].

Numerous alternatively spliced transcripts are elicited in a cell-type-specific manner. Indeed, extensive AS programs are frequent during early embryonic development and function pivotal regulatory roles in cell fate determination and differentiation, organogenesis, and tissue development [5, 6]. The meticulous expression of AS profiles maintains tissue identity and function, while the temporal expression switch between splicing variants usually promotes cell differentiation and tissue development [7]. Importantly, dysregulated AS pathways often employ aberrant biological actions causing various disease conditions in the human body [8].

In this review, we accumulate comprehensive insights into the AS regulatory role in adipogenesis based on the available studies from 2002 to 2020. We put a large emphasis on the reported adipose developmental-stage-specific alternative splicing events (ASEs) and the integrated regulatory role of different splicing regulators during adipogenic differentiation processes in white, brown, and beige adipocytes, respectively. We also explore recent advances in the association of the splicing program with the pathophysiological obese condition and metabolic disorders. In summary, our review is an exclusive rendition of three main aspects: how AS exerts a profound influence on adipocyte development, and how the pathologic roles of alternative RNA splicing variations operate in obesity and related metabolic disorders, and great emphasis on discussing the application of AS in the form of viable therapeutic option for combating obesity and obesity-associated harmful chronic diseases.

### Alternative RNA splicing and adipogenesis

#### Regulatory mechanism of cellular alternative splicing process

**Fig. 1:**
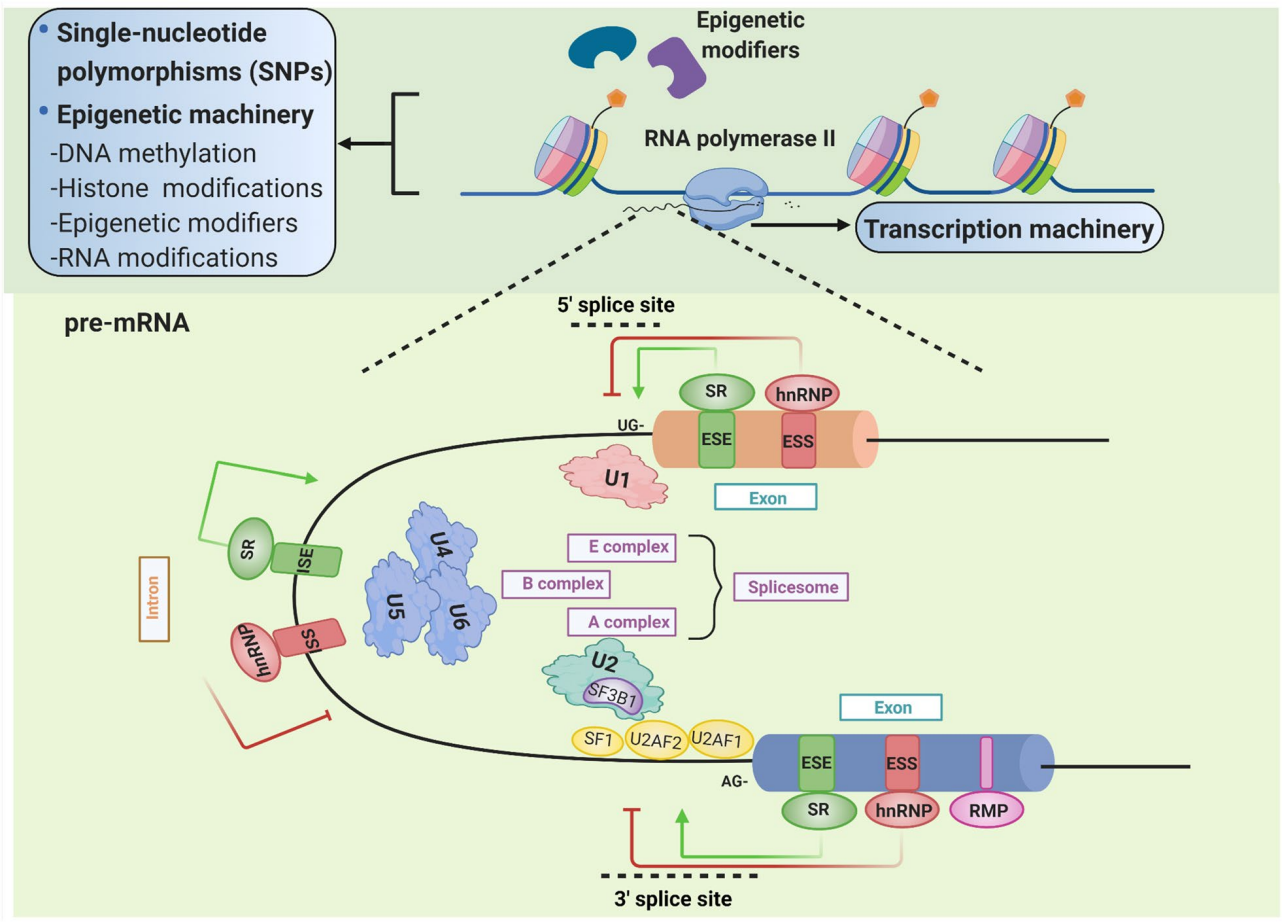
The paradigmatic splicing regulation mechanisms in precursor messenger RNA (pre-mRNA). Generally, AS has complex correlations with splicing factors, transcriptional machinery (RNA polymerase II elongation rates), and epigenetic modifications (histone marks, epigenetic modifiers, and DNA methylation) at the post- and co-transcriptional levels. During AS process, spliceosome, a highly dynamic ribonucleoprotein complex mainly composed of five different small nuclear ribonucleoprotein (snRNP) complexes (U1, U2, and the tri-snRNP U4/U6. U5 structure), can function in splice sites recognition and reaction on pre-mRNA molecules. The spliceosome assembly process starts with the recognition of the initial 5′ splice site by E complex containing U1 snRNP at the GU motif and the identification of 3′ splice site by three interacting proteins-U2 small nuclear RNA auxiliary factor 1 (U2AF1), U2 small nuclear RNA auxiliary factor 2 (U2AF2), and splicing factor 1 (SF1); then the A complex of U2 snRNP binds to the branch site whose key protein component is splicing factor 3 subunit B1 (SF3B1); finally, the U4/U6. U5 tri-snRNP, forming B complex, triggers the core catalytic reaction of spliceosome. During this process, splicing factors target and interplay with spliceosome components to regulate 5′ and 3′ splice-site recognition flanking the alternative exon, such as serine/arginine-rich (SR) proteins and heterogeneous ribonucleoproteins (hnRNPs). The SR proteins are general splicing activators via binding to exonic/intronic splicing enhancers (E/ISEs) to facilitate exon formation, whereas hnRNPs are general splicing inhibitors via binding to exonic/intronic splicing silencers (E/ISSs) to interfere with the splice site recognition

Different RNA splicing modes give rise to different mRNA transcripts with varying coding potential, untranslated regions (UTRs), or RNA stability. Substantially, the pre­mRNA splicing outcomes can be determined under complex mechanisms involving splicing factors, transcriptional machinery, epigenetic modifications, and genomic mutations/single-nucleotide polymorphisms (SNPs) (Fig. 1).

### Splicing-site selection regulatory mechanism

AS decisions are influenced by global interactions between cis­acting RNA sequences and the surrounding sequence contexts (including exonic/intronic splicing enhancers (E/ISEs) or silencers (E/ISSs), 5′ and 3′ splice sites) and their binding trans­regulatory factors (e.g., splicing factors, etc.) [9, 10]. Indeed, RNA-binding proteins (RBPs), accounting for most splicing factors, can exert different regulatory functions in splice-site choices under a cooperative or antagonistic manner through their expression levels, nuclear localization, mRNA stability, and their own splicing regulated by other RBPs. This process usually exhibits a mechanistic interplay between RBPs with other multiple splicing regulators, such as epigenetic and transcriptional machinery, at the post- and co-transcriptional levels. Moreover, the outcome prediction and identification of genome-wide splicing pattern in specific cell conditions have been proposed to be determined by a “splicing code” (large combinations of RNA features in AS regulation), combing cis-regulatory features and splicing factor binding [11], whose depth and complexity remain to be deciphered.

### Epigenetic splicing regulation mechanisms

Histone modifications, epigenetic modifiers, and DNA methylation can function broadly in RNA splice site recognition, spatially and temporally [12–16]. A set of underlying mechanistic modes of particular histone post-translational modifications (hPTMs) in modulating alternative exon splicing (exon inclusion/exclusion) are: (1) through direct recruitment or sequestration of splicing factors [14]; (2) and adaptor proteins [17]; (3) or through indirect transcriptional elongation regulation of the RNA polymerase II (Pol II) [18, 19]. Besides, RNA modifications, such as N6-methyladenosine (m^6^A), also have functional relevance with the AS regulation process [20, 21]. It was first reported that m^6^A peaks exhibited more considerable enrichment in multi-isoform genes than in single-isoform ones and in alternatively spliced exons than in constitutive ones [22]. Subsequent results further revealed the main m^6^A modification-related regulatory mechanisms, involving the binding modulation of splicing factors throughout RNA conformation [23], the binding of m^6^A readers near the splice sites that affects the recruitment of splicing factors [24], and the splicing-factors-mediated recognition of m^6^A motifs [25].

### Other AS regulation mechanisms

Transcription initiation and Pol II elongation rates greatly influence the RBPs’ recognition to nascent pre-mRNA splice sites of various strengths in a time-dependent manner [26–29]. Works have underscored the determinative role of kinetic coupling in co-transcriptional splicing reactions that the upstream site events have a competitive advantage over the downstream, especially when elongation rates are reduced [29, 30]. Under the slow Pol II control, weak splice site recognition is favored, and the alternative up-and down-regulation of exon inclusion can be allowed [28]; by contrast, fast Pol II attenuates weak splice site recognition and promotes alternative exon-skipping events [31].

Additionally, AS patterns could also be tightly affected by genetic mutations [32]. According to the Human Gene Mutation Database, SNPs that fall within critical regions of cis-acting splicing elements and splicing factors may display mis-splicing phenotypes [33]. Of note, 22% of disease-causing SNPs, initially identified as missense mutations, appear to be more likely to disrupt the pre-mRNA splicing process than canonical variations; together with the known splicing mutations category, they suggest that about one-third of disease-causing mutations could affect pre-mRNA splicing patterns [33].

#### Regulatory mechanisms of adipogenesis process

**Fig. 2:**
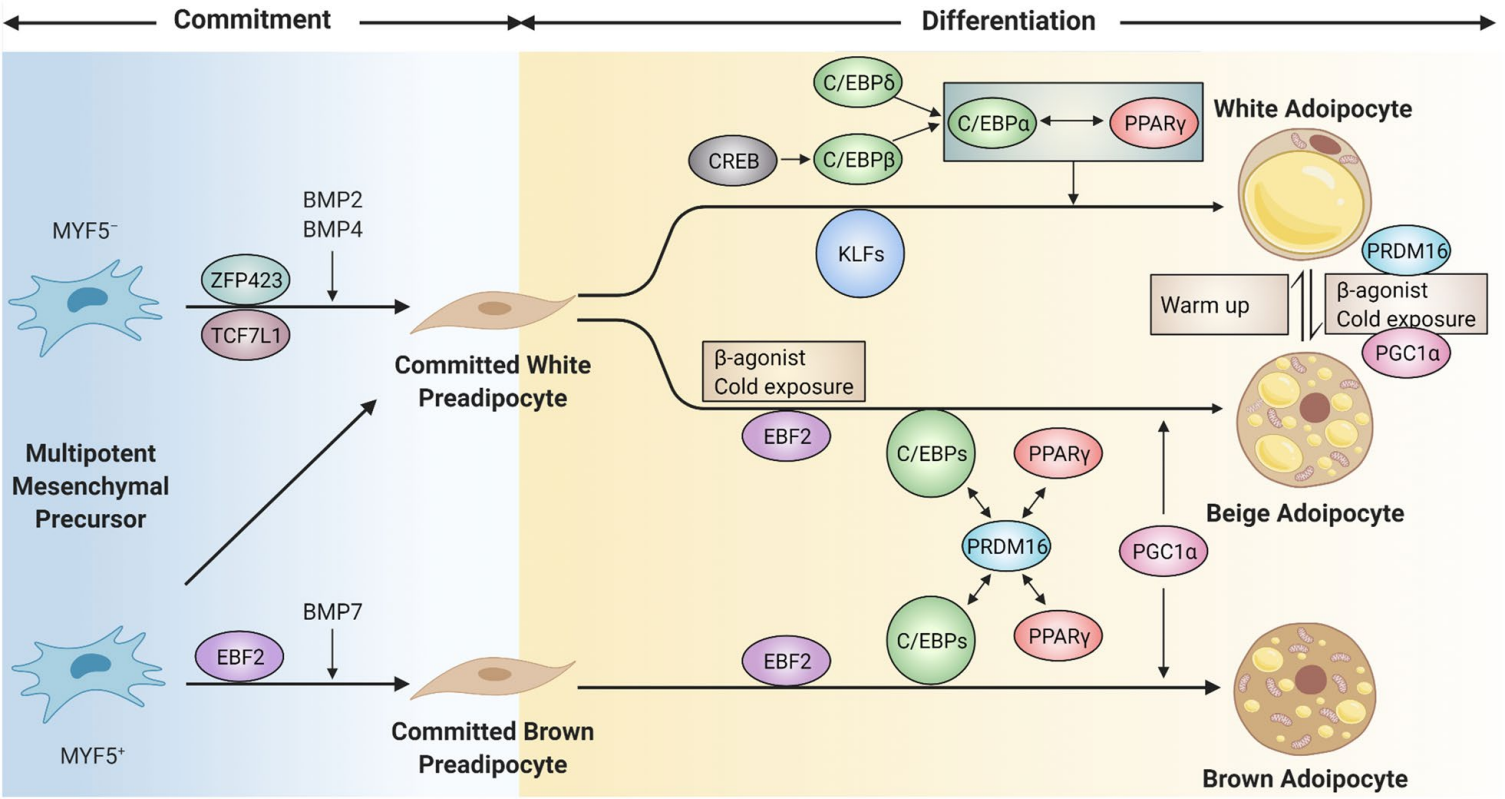
Molecular mechanisms of the two-phase adipogenesis process. A common two-phase adipogenesis process is described: early determination and terminal differentiation phases, involving an intricate integration of cytoarchitecture, transcription factors and co-regulators, and signaling pathways. In the first commitment step, mesenchymal progenitors commit their fate to certain preadipocytes exclusively under the restriction control of bone morphogenetic protein (BMP) signaling. Subsequently, during the second adipogenic differentiation step in both white and brown adipocytes, the master regulator of adipogenesis-peroxisome proliferator-activated receptor-γ (PPARγ) is stimulated and synergizes with CCAAT/enhancer-binding protein α (C/EBPα) to fully activate a transcriptional cascade contributing to and maintaining stable maturation of functional adipocytes and to further engage in adipose biology modulation. Significantly, the zinc-finger transcriptional co-regulator PR domain-containing 16 (PRDM16), cooperating with PPARγ and C/EBPs, and PPARG coactivator 1α (PGC-1α) are of fundamental importance to induce mitochondrial biogenesis and BAT-specific genes expression in the brown adipocyte terminal differentiation and white adipocytes browning process. *MYF5* myogenic factor 5, *BMP2,4,7* bone morphogenetic protein 2,4,7, *EBF2* early B-cell factor 2, *ZFP423* zinc finger protein 423, *TCF7L1* T cell-specific transcription factor 7-like 1, *CREB* cAMP-response element-binding protein, *KLFs* Krüppel-like factors

Adipose tissues, including white adipose tissue (WAT), brown adipose tissue (BAT), and beige adipose tissue, lie in the regulatory center of metabolic functions and systemic energy homeostasis, linking closely with multiple physiological processes, such as lipid metabolism, insulin sensitivity, satiety, thermoregulation, and inflammation [34, 35]. Adipose tissues exhibit high plasticity to undergo a two-phase adipogenesis process (early commitment and terminal differentiation) that multipotent mesenchymal precursors restrict their fate to the committed adipocyte lineage (Fig. 2), surging newly insulin-responsive and profoundly influencing metabolic health and energy homeostasis [36–38].

Distinguished from the WAT adipogenesis that mainly occurs in the postnatal period, BAT mainly develops early during embryonic development before birth [39]. Nevertheless, the nature and origin of active beige adipocytes with improved thermogenesis ability is an intensely ongoing debate. Upon environmental cold or high-fat-diet exposure or sympathetic stimulation, white adipocytes can undergo trans-differentiation into brown-like adipocytes dependent on PPARγ [40, 41], or that dormant beige adipocytes in WAT are recruited and activated [42], or that mesenchyme-derived resident precursors are differentiated de novo [43]. Notably, the process of beige adipogenesis to increase the number of metabolically active adipocytes ameliorating metabolism-related profiles is highlighted as a promising therapeutic strategy for obesity and metabolic disorders.

### Alternative splicing events implicated in adipogenesis of different adipose tissues

Within the past decades, high-throughput RNA sequencing techniques, such as next-generation sequencing (NGS) of RNA, have become popular tools for studying pre-mRNA ASEs through unbiased assessments. These technologies, generating abundant transcriptomic data, help to identify widespread ASEs and also a new class of adipogenesis-specific splicing profiles. Indeed, annotation of different splicing choices and splicing transitions occurred during the time course of adipocyte development is a common theme in deciphering the AS regulatory mechanism in adipogenesis.

#### Alternative splicing events in WAT adipogenesis

**Fig. 3:**
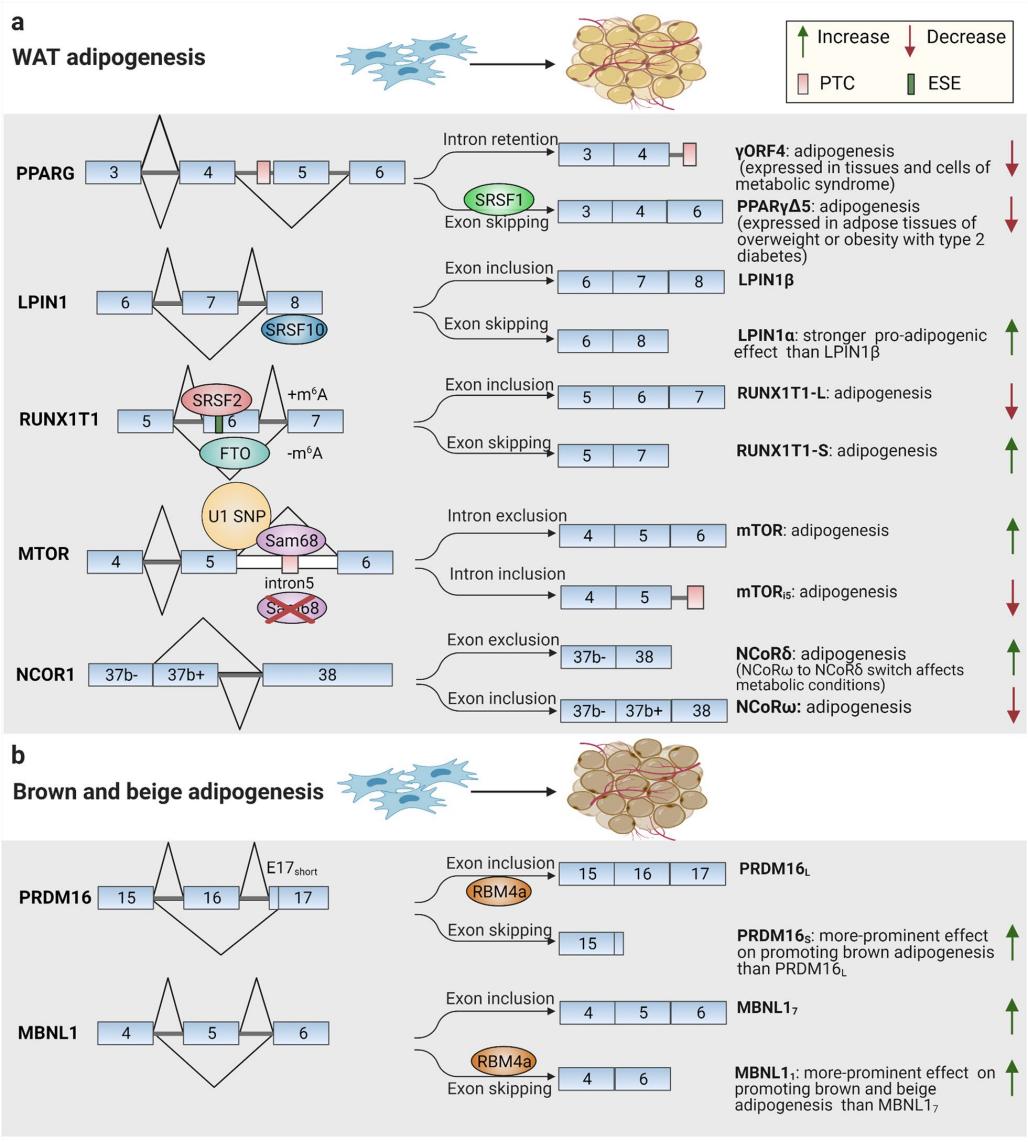
Examples of modulated alternative splicing events implicated in white, brown, and beige adipogenesis

Mechanistic investigations clearly indicate that distinct AS-originated transcripts of critical adipogenic regulators contribute to the regulation of divergent cell fate determination pathways. A typical example is tension-induced/inhibited proteins (TIPs), known stretch-responsive factors with three alternative spliced isoforms-tension-induced/inhibited protein 1 (TIP-1), 2 (TIP-2), and 3 (TIP-3), which function differently in the cell fate selection of adipogenesis or myogenesis [44]. The TIP-3 isoform recruited by PPARγ2 promoter can stimulate the selection into the adipogenic program, while TIP-1 promotes myogenic differentiation [44]. Moreover, the past decades have also profusely addressed AS to extend the action modes of adipogenic regulators that exert different effects on adipocyte terminal differentiation mediated by switches or alterations in splicing patterns, as mainly summarized in Fig. 3a. Among them, some of the splicing switches can appear with slower kinetics in a developmentally regulated manner during adipogenesis, such as protein kinase C βII (PKCβII) [45] and melanocortin 2 receptor (MC2-R) [46]. Further transcriptomic characterization of differentiating human mesenchymal stem cells (hMSCs) has led to the finding of time-specific AS profiles of this process, which reveals multiple AS types in genes of adipogenic regulators, including cassette exon, alternative to 3′ splice site, and alternative to 5′ splice site, and topological distribution patterns on potential key ASEs [47].

**Fig. 4:**
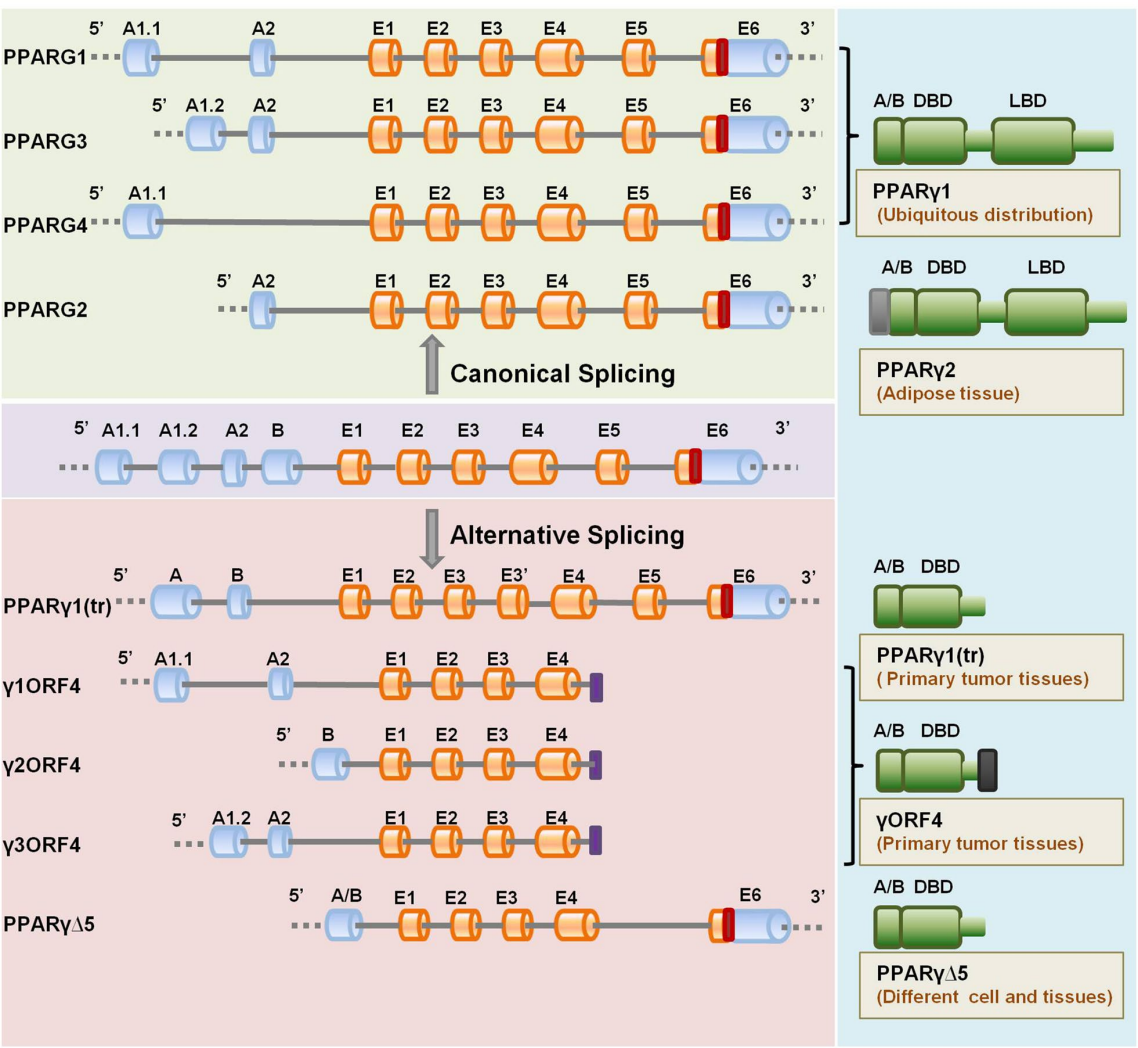
Schematic illustration of canonical and alternative spliced transcripts of peroxisome proliferator activated receptor gamma (PPARG) gene. In the central part, the PPARG gene structure is depicted. Four main PPARG canonical splice transcripts-PPARG1, PPARG2, PPARG3, PPARG3, and PPARG4-and alternative splice variants-PPARγ1(tr), γ1ORF4, γ2ORF4, γ3ORF4, and PPARγ∆5-are shown in the upper and below part, respectively. The corresponding encoded protein isoforms with functional domains are sketched in the right panel with divergent distribution patterns. The orange columns represent exons; the grey lines represent introns; the blue columns represent untranslated regions (UTRs); the grey column in PPARγ2 represents the 30 additional amino acids at N-terminus; the dark column in γORF4 represents the 21 amino acids at C terminus; the red rectangles represent stop codons; the purple rectangles represent premature termination codons (PTCs)

Actually, some of the previously unidentified alternatively spliced variants in specific tissues or cells can be found in extensive analyses of related gene locus through combined approaches of oligonucleotides, cloning for various isoforms, Sanger sequencing, as well as re-analysis on published RNA-sequencing datasets or Genotype-Tissue Expression supported information. Based on these methods, numerous findings exist of the inhibitory effects that splicing alterations in adipogenic genes have on the adipocyte differentiation process. A series of spliced variants in genes of peroxisome proliferator-activated receptor gamma (PPARG) [48–51], preadipocyte factor-1 (Pref-1) [52], and nuclear factor erythroid 2-related factor 1 (NRF1) [53] in adipogenic differentiation offer excellent examples for this case. Above all, for PPARγ, the necessary “master regulator” of adipogenesis, alternative RNA splicing often serves as a dominant-negative mechanism antagonizing PPARγ biological activity and function. Apart from four main canonical splice transcripts of PPARG (PPARG1, PPARG2, PPARG3, and PPARG4), the PPARG gene regions which encode for DNA-binding domain (DBD) and ligand-binding domain (LBD) containing multiple cis-regulatory elements can generate several inhibitory truncated isoforms-PPARγ1(tr) [48], γORF4 [49, 50], and PPARγ∆5 [51]-by alternative promoter usage and splicing processes (Fig. 4). Substantially, in adipose tissues, these alternative PPARG splice variants show differential ligand specificity and functional relevance during adipogenesis: γORF4, in lack of LBD, suppresses adipogenesis in a stage-specific expression manner [49, 50]; PPARγ∆5, another naturally occurring negative isoform, undergoes exon skipping event modulated by serine and arginine rich splicing factor 1 (SRSF1) leading to a lack of the entire LBD and impairs the PPARγ transcriptional network in a negative feedback mechanism during adipogenesis [51] (Fig. 3a). In addition, AS determines Pref-1 function by regulating the production of the biologically negative soluble isoform during adipogenesis. The alternatively spliced transcripts of Pref-1 could suppress adipogenesis in differentiating 3T3-L1 cells either in a juxtacrine/paracrine or in an endocrine manner that Pref-1A and Pref-1B, producing two large soluble forms, can biologically activate anti-adipogenic signaling, whereas Pref-1C and Pref-1D, generating only smaller fragments lacking in-frame juxtamembrane, have no biological effectiveness in adipogenesis [52].

It is worth noting that the programmed AS alterations in transcriptional corepressors can contribute to the conversion in the affinity of target gene panels, transcriptional environment, and biological program during adipogenesis. Such mechanism has been implicated in the nuclear receptor co-repressor 1 (NCoR1)/silencing mediator of retinoid and thyroid hormone receptor (SMRT) [54–56]. Beyond the influence of NCoR1/ SMRT on the adipogenic differentiation phenomenon detected in 3T3-L1 cells of NCoR1/SMRT pan-specific knockdown or site-specific mutagenesis in mice models [57, 58], AS pathway generates a set of correlated splice variants with disparate tissue distribution characteristics and numbers and sequences of receptor interaction domains (RIDs) (associated with different PPARγ affinity in adipocytes) that serve to diversify their transcriptional and biological effects on adipogenic differentiation process [55, 56]. One study overexpressed different NCoR splice variants in 3T3-L1 cells to introduce adipogenesis ex vivo and revealed that NCoRω suppressed, whereas NCoRδ with fewer RIDs accelerated adipogenesis, and the decrease in their relative abundance could promote the adipogenic terminal differentiation [54] (Fig. 3a).

Additionally, the programmed splicing process of apoptotic-resistant factors is a determinate switch in adipogenesis, allowing preadipocytes to differentiate into mature adipocytes of highly pro-survival characteristics [59]. Through the utilization of differentiating preadipocytes in vitro, it was identified that protein kinase C δ (PKCδ), Bclx, and caspase9 could be alternatively spliced to convert to their pro-survival variants, producing dramatic biological effects on adipogenic development [59]; the naturally occurring splice variants-protein kinase C δII (PKCδII) of pro-survival activity and protein kinase C δI (PKCδI) of pro-apoptosis effect-were expressed increasingly and decreasingly, respectively, to facilitate an anti-apoptosis phenotype in mature adipocytes through the Bcl2 pathway [60, 61].

#### Alternative splicing events associated with BAT adipogenesis and beige adipocyte development

During the adipocyte differentiation of brown and beige adipocytes, programmed splicing shifts also exert broad influences on these processes and energy expenditure signatures. Through performing transcriptome analyses in embryonic BATs and postnatal BATs [62], discriminative splicing profiles have been identified, including several adipogenic regulators, such as PRDM16 [63] and muscleblind-like 1 (MBNL1) [64] (Fig. 3b). Regarding the specific splicing transition of PRDM16, the determinant transcriptional regulator in both brown and beige adipocyte development [65], a gradual alternatively spliced isoforms shift from an exon inclusion isoform (PRDM16^+ex16^-PRDM16_L_) to an exon exclusion one (PRDM16^−ex16^-PRDM16_S_) has been proved to contribute to a more prominent effect on the differentiation gene program and energy expenditure in the development of brown adipocytes [63]. Results from co-immunoprecipitation tests identified that the splicing factor RNA-binding motif protein 4a (RBM4a) enabled the regulated splicing switch determining the relative expression of the two PRDM16 transcripts, which could constitute a feed-forward circuit with the RBM4a abundance [63]. Besides, RBM4a also participates in the regulated generation of a splicing mode change of MBNL1 (from MBNL1^+ex5^ isoform 7 to MBNL1^−ex5^ isoform 1) through an autoregulatory mechanism, bringing about a more robust simulation in beige cell-selective splicing program throughout BAT development and during the in vitro beige adipogenesis [64] (Fig. 3b).

However, the AS regulatory mechanism in brown and beige adipocyte development has not been comprehensively investigated. Significant to our understanding is whether AS networks modulate brown and beige adipogenesis through undefined mechanisms and what biological roles they act during this process. Additionally, global transcriptome surveys identifying the spectrum of genes with differentially expressed ASEs at the exonic resolution have been conducted in white adipogenesis, whereas much remains to be completed to characterize precise and extensive genome-wide analysis during brown and beige adipogenesis.

### Splicing regulators involved in adipogenesis

Emerging evidence combined functional mechanistic studies in specific adipocytes, transgenic mice models, and high-throughput methods has extensively identified the physiological roles of distinct RNA splicing regulators in splicing networks for white-, brown-, and beige-adipocyte differentiation and maturation process. Among them, are splicing factors-Src-associated substrate during mitosis of 68 kDa (Sam68), serine and arginine rich splicing factor 10 (SRSF10), and RNA binding motif 4 (RBM4), splicing actions of which function significantly during adipogenesis (Table 1). Moreover, multiple other types of molecules with various biological functions, such as fat mass and obesity-associated (FTO), have been studied and demonstrated to bring about functional consequences of AS in adipogenic regulators. Hence, changes in their target splicing networks often exert a vital influence on adipogenesis process.

**Table 1:** Major splicing regulators involved in adipogenesis processes of white, brown, and beige adipose tissues

Splicing regulators	Phenotypes in transgenic animals	Splicing targets	Splicing effects	References
Sam68	The Sam68-KO(knockout) mice: a lean phenotype with reduced body weight and adiposity; WAT browner; increased thermogenesis; reduced lipid stores in BAT; improved insulin sensitivity; abnormal neuronal processes; defective spermatogenesis and osteogenesis	mTOR; Rps6kb1	Enhance WAT adipogenesis; impair browning trans-differentiation	[66–74]
FTO	1. The FTO-KO mice: increased postnatal lethality; a lean phenotype; postnatal growth retardation; decreased adiposity; increased energy expenditure 2. The FTO-overexpression mice: obesity with an increase in fat mass; hyperphagia; marked glucose intolerance	RUNX1T1	Enhance WAT adipogenesis	[75–79]
SRSF10	The SRSF10-KO mice: multiple cardiac defects; severely impaired WAT development in embryos	LPIN1; PGC-1α	Enhance WAT adipogenesis	[80–82]
RBM4	The RBM4a-KO mice: impaired development of BAT, muscles, and pancreatic β-islets; hyperlipidemia	PRDM16; MBNL1 BAT splicing cascades: RBM4a-SRSF3-MAP4K4; RBM4-MEF2C; RBM4-Nova1-SR-SF6; RBM4-SRPK1; RBM4-Acin1-SRSF3	Enhance BAT adipogenesis	[62, 63, 83–88]

#### Splicing regulators functioning in WAT adipogenesis

The functional AS outcomes of different splicing regulators have been convincingly documented in WAT adipogenesis. Sam68, highly involved in cellular RNA processing events and signal transduction pathways [70, 89], has been demonstrated in adipocyte development to increase the number of early adipocyte progenitors by controlling AS of the mechanistic target of mTOR signaling [66, 68]. The Sam68 binding sites often localize near AS junctions within pre-mRNAs, so they often engage in regulating neighboring alternative exon usage and splice site selection as either splicing enhancers or silencers [69]. In adipocytes, Sam68 can recognize the 5′ splice site of mTOR intron5 through recruitment and interaction with U1 snRNP and bind to intronic splice elements where an in-frame premature termination codon (PTC) exists, which helps to promote an efficient intron exclusion [66, 70]; however, in Sam68 deficiency adipocytes, mTOR intron5 retention occurs, generating a short and unstable transcript degraded by nonsense-mediated decay (NMD), thus causing impaired insulin-stimulated S6 and Akt phosphorylation pathway [66] (Fig. 3a). Additionally, another independent study conducted cross-linking and immunoprecipitation and showed that Sam68 could also counteract the splicing effects of SRSF1 in splicing regulation of ribosomal S6 kinase (Rps6kb1) gene during adipogenesis, resulting in a decrease in the abnormal inhibitory short isoform-Rps6kb-002 [71].

Moreover, another axis linking one splicing factor with specific ASEs and clear functional outcomes in adipocyte development has also been demonstrated. The RBP SRSF10 is an atypical SR protein with distinguished functions whose activity is positively or negatively determined by cycles of phosphorylation and dephosphorylation-SRSF10 acts as a general splicing repressor by dephosphorylation [90], while functions as a sequence-specific splicing activator under phosphorylation conditions [91]. Besides, in controlling exon splicing, SRSF10 functions positively or negatively depending on the binding locations that the binding on cassette exon leads to exon inclusion, while the binding to downstream exon promotes exon skipping [92, 93]. For adipocyte biology, SRSF10 can directly repress exon inclusion in LPIN1, forming an adipogenic exon-skipping isoform LPIN1α to promote initial adipocyte differentiation [80] (Fig. 3a), and in PPARG coactivator 1α (PGC-1α), causing an NMD in the PGC-1α pre-mRNA to affect gluconeogenesis and glucose metabolism [81].

As mentioned above, the evidence lines that splicing machinery and epigenetic modification factors have close interactions can be identified in the adipogenic differentiation modulation process. FTO is the first identified gene providing the strongest genetic association with human non-syndromic obesity [75, 76]. In one study combining transcriptome analyses with m6A-seq, it has been revealed that FTO can mediate m^6^A demethylation mechanism extended in AS process of one adipogenic factor-runt-related transcription factor 1 (RUNX1T1) during adipogenesis [77, 78]. FTO antagonizes the RNA binding ability of another splicing factor-serine and arginine rich splicing factor 2 (SRSF2) and inhibits target exon inclusion through demethylating of m^6^A-RRACHs (R = G or A; H = A, C or U) near exonic splice sites which are spatially overlapped the ESE binding regions of SRSF2 [77] to produce more exon-skipped isoform RUNX1T1-S with pro-adipogenic activity and decrease negative constitutive isoform RUNX1T1-L, thus leading to an active effect on adipocyte differentiation [77] (Fig. 3a).

Of note, accumulating evidence has reported that other types of the molecule and signaling pathway can play roles in splicing decisions through interacting with or controlling the splicing activity of splicing machinery present in adipocytes, which are sufficient to modulate adipogenesis via the relative abundance regulation of adipogenesis-specific isoforms, such as molecular scaffold protein 14-3-3 proteins [94, 95] and zinc finger protein 638 (ZNF638) [96] in the recruitment and sequestration of different trans-acting splicing factors, Clk/STY (cdc2-Like Kinase 1) in proper phosphorylation of serine/arginine-rich protein 40 (SRp40) [45, 97], and long non-coding RNA NEAT1 associated with SRp40 phosphorylation [98].

#### Splicing factors involved in BAT adipogenesis and beige adipocyte development

The spectrum of splicing factors identified in brown adipocyte differentiation process and intricate physiological functions of BAT mainly includes RBM4, serine and arginine rich splicing factor 6 (SRSF6), serine and arginine rich splicing factor 3 (SRSF3), and serine and arginine rich splicing factor protein kinase 1 (SRPK1). Given that the physiological roles of splicing factors functioning in brown- and beige-adipocyte formation processes are recognized still at an early stage, further identification for the underlying mechanism is needed.

The splicing factor RBM4, firstly identified in tissue-specific splicing network of muscles and pancreatic β-islets, can multifunctionally participate in post-transcriptional regulation and lies in the center hub of AS decisions in brown and beige fat development and functions (Table 1). The ablation of RBM4a in mice (RBM4a^−/−^) has been found to cause impaired development of interscapular BAT and abnormal triglyceride clearance in serum [84]. Moreover, in RBM4-deficiency or-overexpression adipocytes where gene-splicing profiles are reprogrammed, the expression level of BAT development inhibitors (neuro-oncological ventral antigen 1 (Nova1), polypyrimidine tract-binding protein 1 (PTBP1), and 2 (PTBP2)) or adipogenic factors (insulin receptor (IR), PPARγ, Pref-1, fibroblast growth factor receptor 2 (FGFR2), PRDM16, and bone morphogenetic protein 7 (BMP7)) can be affected differently [84, 85], modulating brown and beige adipogenesis, mitochondrial activity, and energy expenditure process. In particular, RBM4 mostly interacts with intronic splicing regulatory elements and inhibits exon inclusion, functioning either as a splicing activator or an inhibitor in different genes [99]. Further, a set of RBM4-governed splicing cascades are indicated through deep RNA-sequencing during the differentiation of brown adipocytes, including RBM4-PRDM16 network [63], RBM4-Nova1-SRSF6 pathway [62], RBM4a-SRSF3-mitogen-activated protein kinase kinase kinase kinase 4 (MAP4K4) pathway [83], RBM4-myocyte enhancer factor 2c (MEF2C) [86], RBM4-SRPK1 [87], and RBM4-apoptotic chromatin condensation inducer 1 (Acin1)-SRSF3 [88], generating different splice isoforms to function distinct roles in transcriptional regulation and form feedback circuits associated with RBM4 abundance.

### Alternative splicing machinery has correlations with obesity and metabolic disorders

**Fig. 5:**
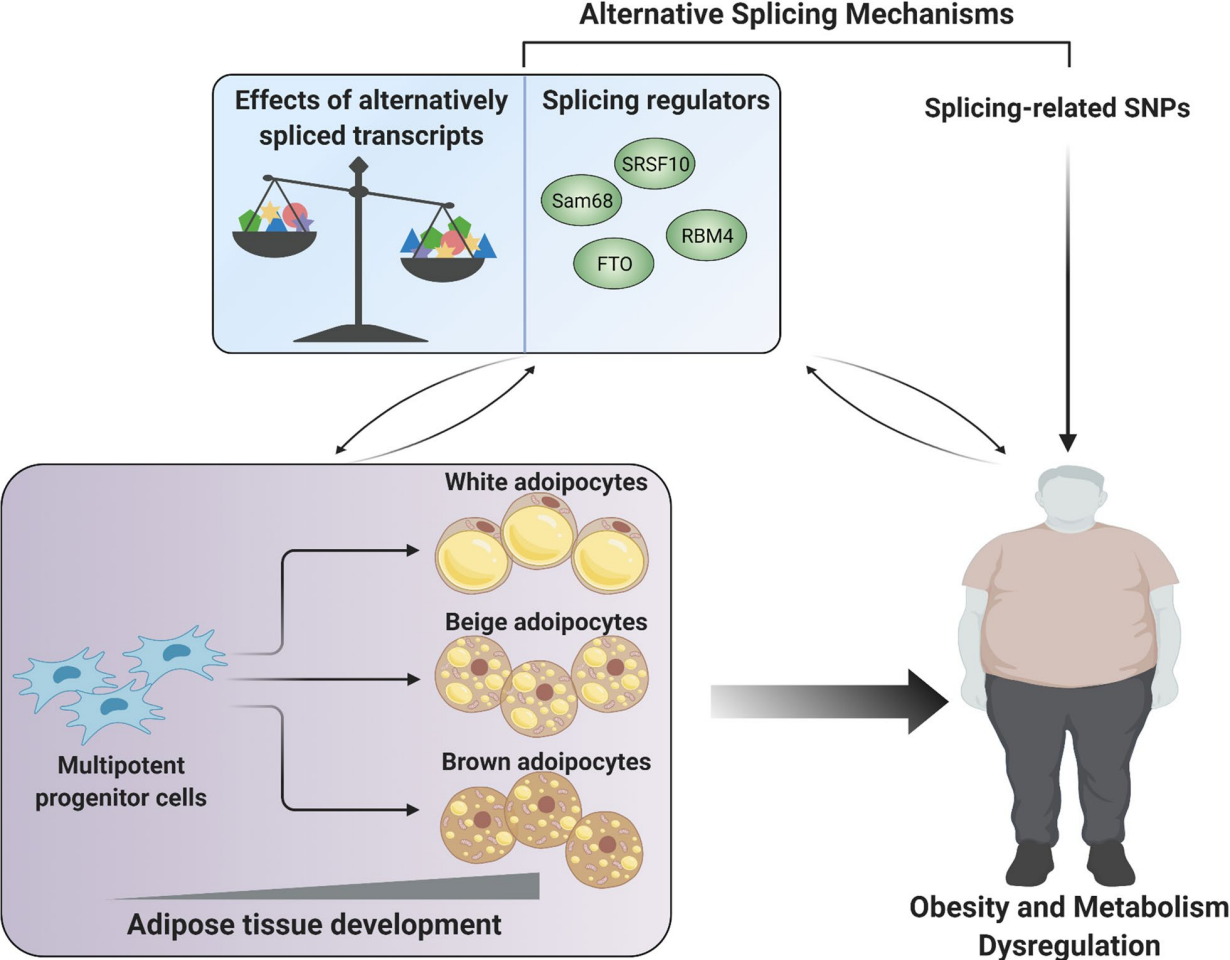
Schematic diagram of alternative splicing mechanism in adipogenesis and metabolic health

Obesity and overweight, a severe growing health concern worldwide, dramatically elevates human mortality risk factors along with different comorbidities, such as metabolic syndrome, type 2 diabetes, cardiovascular disorders, dementia, and cancers [100]. As reviewed in detail below, emerging evidence constitutes a mechanistic understanding that both alternative mRNA mis-splicing patterns and aberrant expression or splicing efficiency of splicing factors, contributing to transcriptome changes, have profound associations with human obesity and metabolic disorders, as illustrated in Fig. 5.

#### Aberrant splicing landscapes associated with obesity and metabolic diseases

First and foremost, accumulating evidence has revealed that a set of adipogenesis regulators exhibit abnormal splicing patterns in adipose tissues from obese patients compared with control subjects, suggesting a vital link of AS with adiposity and metabolic phenotypes-usually the susceptibility to extreme body weight gain and serious metabolic illness [50, 51, 101]. One classical example is PPARγ, the master modulator in whole-body crosstalk of metabolic organs, whose canonical transcripts and alternatively spliced negative isoforms exhibit remarkably different enrichment patterns in overweight or obese patients with diabetes: in subcutaneous adipose tissue, the specific expression of PPARγΔ5, a naturally occurring negative PPARG isoform, can display a positive correlation with body mass index (BMI) in the patient’s population of these diseases [51]; γORF4 with dominant-negative activity has also been found to be physiologically expressed in tissues related to complications of metabolic syndrome in humans [50]. Another example involves apoptosis-pathway genes, which can undergo crucial splicing switches to generate prosurvival splice variants (PKCδ, Bclx, and caspase9), promoting the shift to apoptosis-resistant mature adipocytes; while a series of alternatively spliced transcripts are abnormally overexpressed in obese patient-derived adipose tissues [59]. Moreover, other cohort studies revealed quantitative AS changes in the transcriptome in WAT where gene splicing profiles displayed abnormal features in the contexts of nutritional changes, obesity, and dysmetabolism [102], including the genes of Lamin A/C (LMNA) [103], cannabinoid type I receptor (CB1R) [104], and T cell-specific transcription factor 7-like 2 (TCF7L2) [105]. Importantly, their splicing alterations in WAT suggest strong associations with the adipose tissue biology and related metabolic parameters of obesity and type 2 diabetes, such as insulin action, fatty acid metabolism, and systemic and local chronic inflammation. Besides, not limited to adipose tissues, the splicing patterns have also been found to be influenced by hormonal and nutritive events (dietary carbohydrates changes) as well as obese and metabolic states [106], for example, the IR gene in insulin target tissues [107]. Nevertheless, the underlying physiology relevance between these molecular profiles and obesity remains unexplained, thus it will be of particular importance to further investigate and decipher the detailed mechanisms.

Significantly, several groups also identified that imbalances of different splice isoforms could directly lead to the dysfunction of white- and brown-adipose tissues as well as the initial development of diabesity and metabolic disturbances [107–109]. Studies used the mouse mutant engineered to express one particular splice variant of genes, for example, the PGC-1α gene and the synaptosomal-associated protein of 25 kDa (SNAP-25) gene [108, 109], through knockout/knockin replacement, and revealed how these different splice variants functioned in adipose biology of WAT and BAT as well as energy and metabolism homeostasis under the HFD condition, and what effect they exerted on the susceptibility and predisposition to obesity and metabolic diseases. Further, given the heterogeneity and complexity of these related disorders in humans, particularly noteworthy is that evaluating whether and how splicing profile changes have correlations with different obese and dysmetabolic features among patient’s population should be based on seriously comprehensive analysis with different factors, mainly involving the matched age, target tissues, other complications, and medical therapies.

Finally, it may also need to concern that obesity and related chronic metabolic inflammation link intimately to unfolded protein response (UPR) and interfere with endoplasmic reticulum (ER) homeostasis, where the non-conventional splicing process of the X-box–binding protein 1 (XBP1) mRNA mediated by the ER sensor-inositol requiring enzyme 1α (IRE1α) is disrupted [110]. This defective IRE1α-mediated XBP1 splicing process has been proved in the liver in conditions of both genetic and diet-induced obesity; and these ER-remodeling-associated interactions are worth further examination in adipocytes in the obesity setting.

#### Connection of splicing regulators in obesity and metabolic dysregulation

Representative works have demonstrated that splicing machinery components can be associated with the adipogenesis process and further energy expenditure regulation and body metabolic state. In fact, the conditions of dysregulated metabolic state or high-fat diet can bring about altered expression of genes encoding trans-acting splicing factors [102], or alterations in splicing activity [111], both contributing to widespread mis-spliced pre-mRNAs; and abnormal splicing factors-mediated AS program can also contribute to the development of metabolic disorders and diet-induced obesity. For example, the obese and metabolic impact on the reduced expression of transformer 2β homolog (TRA2B/SFRS10) which belongs to the SR-like protein family of splicing factors was observed in liver and muscle tissues from obese patients; the SFRS10 down-regulation can alter the splicing pattern of LPIN1 to induce lipid accumulation, which therefore causes aberrant metabolic phenotypes and obesity [112]. In another example, the vital role of neuro-oncological ventral antigen (NOVA) splicing factors in the pathogenesis of obesity has been confirmed in the NOVA-deficient mice that NOVA-regulated splicing program participates in glycemia increase and thermogenesis suppression in adipocytes [102].

Apart from these expression changes associated with metabolic phenotypes, polymorphisms in the splicing regulator genes detected by amounts of genome-wide association studies in humans, such as FTO, could consistently exert a strong influence on body fat mass and the risk of developing diabesity and type 2 diabetes [113–115].

#### Splicing-related SNPs contribute to obesity and metabolic disorders

Genetic association studies that detect the link of SNPs affecting splicing-related process with obesity and metabolic disorders have made it possible to provide candidate diagnostic tools and potential targets for clinical intervention. Evidence from the sequencing-based approach and large-scale analysis in obese populations has highlighted that the SNPs connected with the aberrant splicing patterns in obesity-associated genes could represent possible biomarkers for extreme BMI susceptibility [116]. Substantially, broad obesity-related mutation spectrums of synonymous and nonsynonymous SNPs are located near splice junctions and are prone to affect the cis-regulatory elements of exons with weak splice sites allowing the shift from constitutive exon patterns to alternative ones and thus give rise to multiple RNA isoforms [116]. Moreover, collective evidence has displayed the tight link between splicing related SNPs and human body metabolic state, involving the genes of uncoupling protein-3 (UCP3) [117], glucose-6-phosphatase catalytic unit 2 (G6PC2) [118], insulin-like growth factor 2 (IGF2) [119], adenylate cyclase 3 (ADCY3) [120], and an intragenic variant of insulin gene (IVS1-6A/T (-23HphI ±)) [121]. For example, a novel genetic variation with a disruption in canonical splice-site acceptor of the IGF2 gene among the Mexican population has been identified to function as a protective factor to reduce the type 2 diabetes risk at ∼ 20% through repressing splicing between the exons 1 and 2 and reducing the expression of IGF2 isoforms [119]. Additionally, one loss-of-function genetic variant of ADCY3 with a disruption of a splice acceptor site causing exon skip or intron retention was detected among the Greenlandic population and positively correlated with increased adiposity and predisposition of type 2 diabetes [120]. Besides, one genome-wide association study among European women has evaluated the physiological relevance of the polymorphisms in the leptin receptor (LEPR) in human diabetes-related traits, which appear to modulate the expression level of plasma soluble leptin receptor-one alternative isoform of LEPR gene correlated inversely with BMI and diabetes risk factors [122].

## Conclusions and future perspectives

Findings provide the mechanistic role of AS in multiple facets of adipocyte development and function and the broad influences on metabolic health. A variety of research efforts have compiled the comprehensive crosstalk between AS and the adipocyte differentiation processes of different adipose tissues and whole-body energy and metabolic homeostasis. Substantially, profiling alternative splicing alterations in obesity and metabolic disorders might provide possible biomarkers and designs for novel diagnostic strategies. Noteworthy, high-throughput RNA sequencing techniques and transcriptome bioinformatics analysis fully support the identification of the massive ASEs amounts and the regulatory role of splicing factors during adipogenesis and facilitate sufficient clarity into how adipogenesis is modulated at different stages on a genome-wide scale.

Nevertheless, our understanding of the AS mechanism involved in brown and beige adipogenesis is still evolving. Further work on deciphering “splicing code” in brown and beige adipogenic differentiation to offer broader and more meticulous insights into the complex interplay of trans-splicing regulators with cis-acting elements is vital for an in-depth understanding of adipocytes physiology. Additionally, more investigations are required on the functional mechanism of specific splice variants from different sorts of adipose tissue in the pathogenesis of obesity. Accordingly, fixing splicing dysregulation emerges as a promising therapeutic option and provides more viable strategies for future management for obesity and metabolic disorders.

## Data Availability

Not applicable.

## Abbreviations

Acin1: Apoptotic chromatin condensation inducer 1
ADCY3: Adenylate cyclase 3
AS: Alternative splicing
ASE: Alternative splicing event
BAT: Brown adipose tissue
BMI: Body mass index
BMP: Bone morphogenetic protein
BMP7: Bone morphogenetic protein 7
CB1R: Cannabinoid type I receptor
Clk/STY: Cdc2-like kinase 1
CREB: CAMP-response element-binding protein
C/EBPα: CCAAT/enhancer-binding protein α
DBD: DNA-binding domain
EBF2: Early B-cell factor 2
ER: Endoplasmic reticulum
E/ISEs: Exonic/intronic splicing enhancers
E/ISSs: Exonic/intronic splicing silencers
FGFR2: Fibroblast growth factor receptor 2
FTO: Fat mass and obesity-associated
G6PC2: Glucose-6-phosphatase catalytic unit 2
hMSCs: Human mesenchymal stem cells
hnRNPs: Heterogeneous ribonucleoproteins
hPTMs: Histone post-translational modifications
LBD: Ligand-binding domain
IGF2: Insulin-like growth factor 2
IR: Insulin receptor
IRE1α: Inositol requiring enzyme 1α
KLFs: Krüppel-like factors
LBP: Ligand-binding domain
LEPR: Leptin receptor
LMNA: Lamin A/C
MAP4K4: Mitogen-activated protein kinase kinase kinase kinase 4
MBNL1: Muscleblind-like 1
MC2-R: Melanocortin 2 receptor
MEF2C: Myocyte enhancer factor 2c
m^6^A: N6-methyladenosine
MYF5: Myogenic factor 5
NCoR1: Nuclear receptor co-repressor 1
NGS: Next-generation sequencing
NMD: Nonsense-mediated decay
NOVA: Neuro-oncological ventral antigen
Nova1: Neuro-oncological ventral antigen 1
NRF1: Nuclear factor erythroid 2-related factor 1
PGC-1α: PPARG coactivator 1α
PKCβII: Protein kinase C βII
PKCδ: Protein kinase C δ
PKCδI: Protein kinase C δI
PKCδII: Protein kinase C δII
Pol II: RNA polymerase II
PPARG: Peroxisome proliferator-activated receptor gamma
PPARγ: Peroxisome proliferator-activated receptor-γ
PRDM16: PR domain-containing 16
pre-mRNA: Precursor messenger RNA
Pref-1: Preadipocyte factor-1
PTBP1: Polypyrimidine tract-binding protein 1
PTBP2: Polypyrimidine tract-binding protein 2
PTC: Premature termination codon
RBM4: RNA binding motif 4
RBM4a: RNA-binding motif protein 4a
RBPs: RNA-binding proteins
RIDs: Receptor interaction domains
Rps6kb1: Ribosomal S6 kinase
RUNX1T1: Runt-related transcription factor 1
Sam68: Src-associated substrate during mitosis of 68 kDa
SF1: Splicing factor 1
SF3B1: Splicing factor 3 subunit B1
SMRT: Silencing mediator of retinoid and thyroid hormone receptor
SNAP-25: Synaptosomal-associated protein of 25 kDa
SNPs: Single-nucleotide polymorphisms
snRNP: Small nuclear ribonucleoprotein
SR: Serine/arginine-rich
SRPK1: Splicing factor protein kinase 1
SRp40: Serine/arginine-rich protein 40
SRSF1: Serine and arginine rich splicing factor 1
SRSF2: Serine and arginine rich splicing factor 2
SRSF3: Serine and arginine rich splicing factor 3
SRSF6: Serine and arginine rich splicing factor 6
SRSF10: Serine and arginine rich splicing factor 10
TCF7L1: T cell-specific transcription factor 7-like 1
TCF7L2: T cell-specific transcription factor 7-like 2
TIP: Tension-induced/inhibited protein
TRA2B: Transformer 2β homolog
UCP1: Uncoupling protein 1
UCP3: Uncoupling protein 3
UPR: Unfolded protein response
UTRs: Untranslated regions
U2AF1: U2 small nuclear RNA auxiliary factor 1
U2AF2: U2 small nuclear RNA auxiliary factor 2
WAT: White adipose tissue
XBP1: X-box–binding protein 1
ZFP423: Zinc finger protein 423
ZNF638: Zinc finger protein 638
